# Evaluating the microcystin-LR-degrading potential of bacteria growing in extreme and polluted environments

**DOI:** 10.1007/s00203-023-03554-4

**Published:** 2023-05-02

**Authors:** Thabile Lukhele, Titus Alfred Makudali Msagati

**Affiliations:** Institute for Nanotechnology and Water Sustainability, College of Science Engineering and Technology, University of South Africa, Florida Science Campus, Johannesburg, South Africa

**Keywords:** Bioremediation, Biolog MT2 assay, Environmental contaminants, Cyanobacteria

## Abstract

Inhabitants of extreme and polluted environments are attractive as candidates for environmental bioremediation. Bacteria growing in oil refinery effluents, tannery dumpsite soils, car wash effluents, salt pans and hot springs were screened for microcystin-LR biodegradation potentials. Using a colorimetric BIOLOG MT2 assay; *Arthrobacter* sp*.* B105*, Arthrobacter junii, Plantibacter* sp*.* PDD-56b-14*, Acinetobacter* sp*.* DUT-2*, Salinivibrio* sp*.* YH4, *Bacillus* sp.*, Bacillus thuringiensis* and *Lysinibacillus boronitolerans* could grow in the presence of microcystin-LR at 1, 10 and 100 µg L^−1^. Most bacteria grew optimally at 10 µg L^−1^ microcystin-LR under alkaline pH (8 and 9). The ability of these bacteria to use MC-LR as a growth substrate depicts their ability to metabolize the toxin, which is equivalent to its degradation. Through PCR screening, these bacteria were shown to lack the *mlr* genes implying possible use of a unique microcystin-LR degradation pathway. The study highlights the wide environmental and taxonomic distribution of microcystin-LR degraders.

## Introduction

Over the years, there has been increasing interest in extreme environments and their inhabitants, referred to as extremophiles (Kochhar et al. 2022). These environments have been shown to support diverse and specialized microbial communities with unique physiologies and metabolism. As means of survival, extremophiles synthesize unique enzymes and protective biomolecules with enhanced stability over a wide spectrum of extreme environmental factors (Salwan and Sharma 2022). Therefore, some of these enzymes and biomolecules have found applications in industrial and medical biotechnology owing to their enhanced activity and stability (Mesbah 2022). Inhabitants of highly polluted environments develop physiological traits to withstand and metabolize the contaminants for their survival, and their action contributes to environmental bioremediation (Brito et al. 2013; Sheik et al. 2012). Therefore, these organisms qualify for potential applications in the bioremediation of toxic pollutants from the environment (Raddadi et al. 2015; Sibanda et al. 2017).

**Fig. 1:**
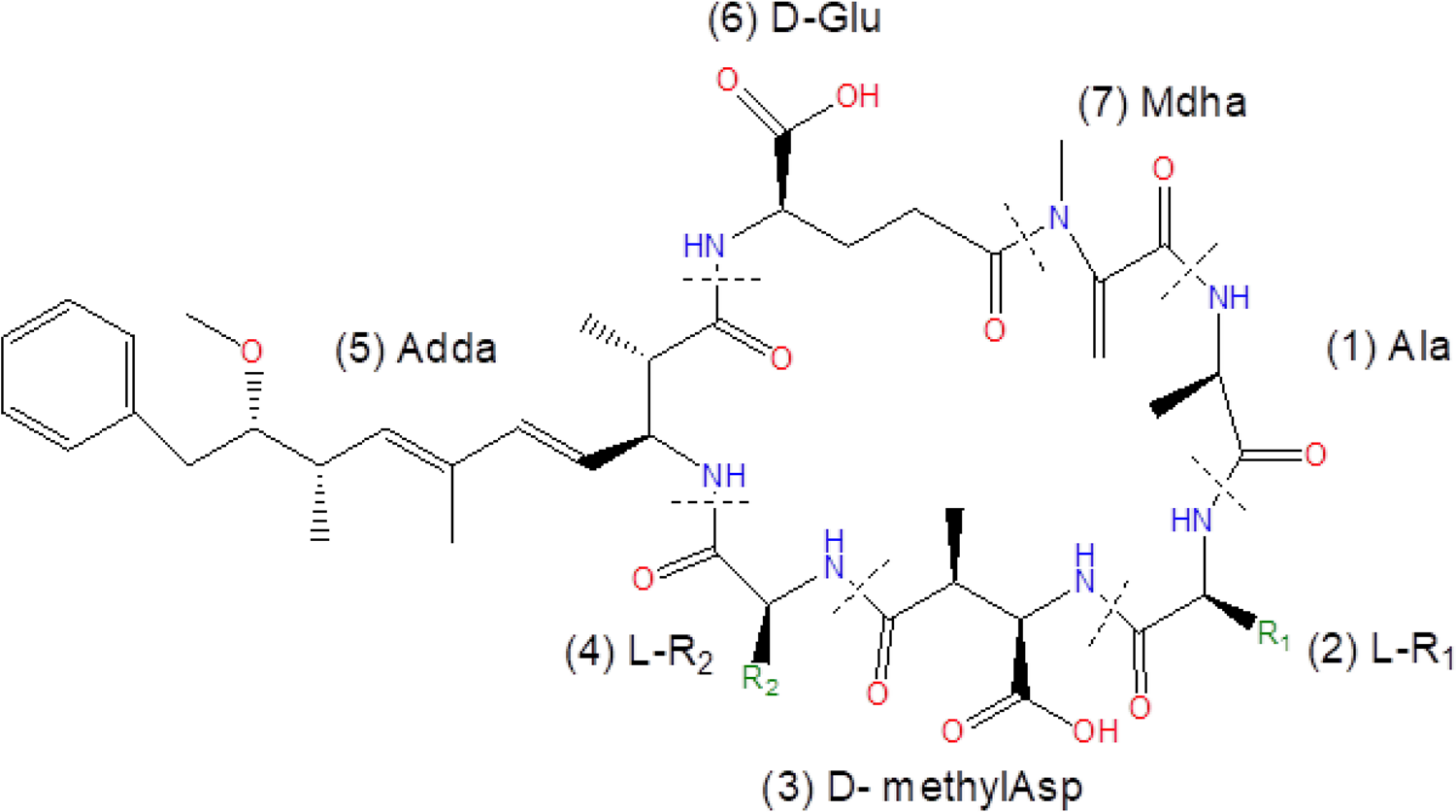
The general chemical structure of microcystins consisting of 7 amino acids arranged into a cyclic structure. Positions 2 and 4 are occupied by variable L-amino acids (Leucine and Arginine for microcystin LR). At positions 5 and 7 are two unique amino acids: Adda (3-amino- 9S- methoxy-2, 6, 8S-trimethyl-10-phenyldeca-4, 6-dienoic acid) and Mdha (methyl dehydro-alanine) which are responsible for the toxicity (Pearson et al. 2010)

A group of toxic environmental contaminants of emerging concern, that pose a risk to human and animal health are microcystins, produced by several groups of freshwater cyanobacteria (Pham and Dang 2019). These are highly toxic compounds with hepatotoxic and carcinogenic effects (WHO 2020). They are cyclic heptapeptides (about 200 variants) characterized by a shared general structure as shown in Fig. 1. Microcystin-LR (MC-LR) is the most toxic microcystin congener which has a lethal dose 50 (LD_50)_ of 50 µg kg^−1^ in mice (Bouaïcha et al. 2019). Due to their cyclic structure, microcystins are very stable at ambient water parameters hence after release from cells they remain in the aquatic ecosystem for long periods with possible food web transfer to terrestrial ecosystems (Pham and Utsumi 2018). Despite their high stability, microcystins are susceptible to microbial degradation, and it serves as the primary mechanism of detoxification and removal in the natural environment (Gagala and Mankiewicz-Boczek 2012). Therefore, there has been a drive to scout for effective microcystin degraders that can be used in potable water treatment (Dziga et al. 2013; Kumar et al. 2019). Although the search has been extended to various environments, extreme and polluted environments remain unexplored. Therefore, the aim of this study was to evaluate the microcystin-degrading potential of bacteria inhabiting extreme and polluted environments.

## Materials and methods

### Materials and reagents

A pure standard of MC-LR (purity ≥ 95%) (Enzo Biochem, Inc., New York, USA) was supplied by BIOCOM Africa, in a lyophilized form. A stock solution of 10 mg L^−1^ was prepared in methanol (LC–MS grade) and divided into small aliquots kept in amber vials kept at – 20 °C. Prior to use in the degradation assays, the standard was filter sterilized using 0.22 µm syringe filters. All growth media were prepared from microbiology, molecular or cell culture grade reagents purchased from Sigma-Aldrich (Sandton, South Africa). Deionized water was obtained from an inhouse water purification system (Milli-Q, Merck Millipore, NY, USA). Biolog^®^ MT2 microplates (Biolog, Hayward, California) were supplied by Anatech South Africa.

### Bacterial strains

This work evaluated the degradation potential of bacterial isolates obtained from different extreme (saltpans, hot spring) and polluted (car wash effluent, oil refinery plant effluent and tannery dumpsite soil). Bacteria from saltpans, hot springs and car wash effluent (Table 1) were isolated in previous works (Selvarajan et al. 2017, 2018; Sibanda et al. 2017). These were received in frozen form and were revived by storing at 4 °C overnight. These were further subcultured in nutrient broth (NB) (3 g yeast extract, 5 g peptone, 5 g sodium chloride, dissolved in 1 L deionized water) and their purity confirmed by plating on nutrient agar (NA) (3 g yeast extract, 5 g peptone, 5 g sodium chloride, 15 g agar, dissolved in 1 L deionized water). Bacteria from the tannery dumpsite soils and oil refinery plant effluent were isolated in this current study.

**Table 1:** List of bacteria obtained from extreme and polluted environments, evaluated for MC-LR-degrading potential using the Biolog MT2 microplate assay

Isolation source	Code	Identity	Gen bank accession no
Hot spring	BR1	*Acinetobacter pittii*	KT748635.1
	BR2	*Acinetobacter* sp. DUT-2	CP014651.1
	BL	*Albidiferax* sp*.* 7B-211	KF441658.1
	MO2	*Acinetobacter junii* strain KMDH2	KU844044.1
	TS1	*Plantibacter* sp*.* PDD-56b-14	KR922102.1
	TS2	*Arthrobacter* sp*.* B105	KJ191026.1
Car wash effluent	CAC11	*Aeromonas tecta* strain L47	KU179360.1
	CAL2	*Shewanella* sp. LH8	KT424972.1
	S1	*Bacillus aryabhattai* strain L54	KU179348.1
	SAS8	*Pseudomonas protegens*	LN995720.1
Salt pan	SPS1	*Thalassobacillus devorans* HME8790	KC134359.1
	SPS2	*Halobacillus* sp. K13	KT353097.1
	SPS6	*Halobacillus* sp*.* A381	JX415307.1
	SPB8	*Halobacillus alkaliphilus* MGR92	KF151860.1
	SPS10	*Salinivibrio* sp. YH4	KR870826.1

### Sample collection

For bacteria isolation grab water samples (effluents) were collected from a wastewater treatment plant of an oil refinery plant and soil was collected from a dumpsite that receives solid tannery waste. Samples were collected using standard aseptic techniques, preserved on ice, transported to the laboratory, and processed not later than 12 h after collection. Prior to isolation, the bacterial communities were acclimatized in MC-LR to enrich heterotrophic MC-LR-degrading populations.

### Enrichment

Soil samples were mixed with sterile 0.85% (w/v) sodium chloride solution (0.85 g sodium chloride dissolved in 100 mL distilled water) and shaken at 160 rpm for 15 min. The supernatant was collected, and 5 mL inoculated into 50 mL sterile NB. For the oil refinery plant effluent samples, 5 mL volumes were inoculated into 50 mL NB. All cultures were incubated at 30 °C with shaking (150 rpm) for 72 h. Subsequently, cells were harvested from the 72 h-old cultures by centrifugation (3500 rpm, 10 min) and washed with phosphate buffered saline (PBS) [137 mM NaCl, 2.7 mM KCl, 8 mM Na_2_HPO_4_, 2 mM KH_2_PO_4_]. The washing was repeated three times after which cells were incubated in PBS for 24 h to deplete any traces of carbon. Cells were then washed with PBS and reconstituted in minimal salts media MSM (pH 7.4) (5 g NaCl, 1 g K_2_HPO_4_, 1 g NH_4_H_2_PO_4_, 1 g (NH4)_2_SO_4_, 0.2 g MgSO_4_. 7H_2_O and 3 g KNO_3_ all dissolved in 1 L deionized water). Microbial suspensions were adjusted to an optical density (OD_590_) of 1.5, and 1 mL was inoculated into 10 mL of MSM spiked with MC-LR. The final concentration of MC-LR in the cell culture was set at 3 mg L^−1^. The cultures were incubated in the dark at 25 °C with shaking (150 rpm) for 21 days. Experiments were set up in duplicate and two negative controls; bacterial cells in MSM with no MC-LR and MC-LR solution with no bacterial cells were included.

### Isolation of probable MC-LR degraders

After 21 days of MC-LR enrichment, surviving bacteria were separated and isolated using standard isolation techniques. Briefly, enrichment culture (100 µL) was spread on NA plates and incubated at 25 °C in a humid chamber. Once growth was visible, the resultant cultures were sub-cultured on NA several times until axenic cultures with varying colony characteristics were obtained.

### Characterization of isolated bacteria

The isolates were characterized by 16S rRNA gene sequence analysis and Matrix-Assisted Laser Desorption Time of Flight Mass spectrometry (MALDI-TOF–MS) biotyping.

#### Matrix-Assisted Laser Desorption/Ionization Time of Flight Mass Spectrometry (MALDI-TOF MS) Biotyping

For MALDI-TOF–MS biotyping, cell lysates were prepared from 24 h old cultures using the formic acid extraction method (Emami et al. 2016). All reagents used for the MALDI-TOF -MS analysis were of analytical reagent grade. For each isolate, a single colony was picked and mixed with 300 µL sterile deionized water. Absolute ethanol (900 µL) was added to the bacterial suspension and mixed by vortexing. Thereafter, the mixture of bacterial cells in ethanol was centrifuged at 15 000 rpm for 2 min and the supernatant discarded. This was repeated until all the ethanol was removed. The pellet was then dried at room temperature for 3 min after which 80 µL formic acid (70% v/v) was added and the mixture vortexed. An equal volume of acetonitrile was added to the mixture and centrifuged at 15 000 rpm for 2 min. The supernatant (1 µL) was drawn and spotted onto a polished steel MALDI target plate (Bruker Daltonics). The spotted lysates were left to dry at room temperature for 1 h prior to overlaying with 1 µL of a MALDI matrix. The matrix used was a saturated solution of α-cyano-4-hydroxycinnamic acid (HCCA) in 50% (v/v) acetonitrile and 2.5% (v/v) trifluoroacetic acid.

### Sample analysis

Samples were analyzed on a Bruker ultrafleXtreme MALDI Biotyper 2.0 (Bruker Daltonics, Germany) with an in-built Flex control software. Spectra were analyzed in a mass to charge (m/z) ratio of 2000–20 000 Da. Data analysis was performed using the MALDI Biotyper Real-time Classification (MBT RTC) software (Bruker^®^). For isolate identification, the following score cut-off values proposed by the manufacturer were used: (≥ 2.000 for species-level, 1.700 to 1.999 for genus level and < 1.700 no reliable identification. Correct species identification was accepted if ≥ 2 out of 4 spectra scored ≥ 2.0 (MALDI Biotyper 3.1 User Manual, Bruker Daltonics, 2012).

#### 16S rRNA gene sequencing

The 16S rRNA genes were amplified by colony PCR using the 27F and 1492R universal primer pair (Chakravorty et al. 2008). Genomic DNA was extracted from 24-h-old cultures using the heating method. For each isolate, a single colony was picked with a 10-µL pipette tip and mixed with 10 µl nuclease free water. Then, the bacterial suspension was heated in a heating block at 96 °C for 10 min. The mixture was centrifuged at maximum speed for 30 s and the supernatant drawn and kept in a separate tube for use in subsequent reactions. PCR was performed in a 25 µL reaction mixture containing 9.5 µL sterile nuclease free water, 12.5 µL 2X PCR master mix (Qiagen Top *Taq*), 0.5µL each of forward and reverse primer and 2 µL of the bacterial DNA suspension. PCRs were performed in a BioRad T100 thermal cycler using a protocol previously described in Selvarajan et al. (2018). Successful amplification was confirmed with gel electrophoresis. Ethidium bromide stained 1% agarose gels in 1 × TAE buffer were run at 80 V for 90 min. Gels were viewed under a Gel Doc imager (Bio Rad). Resultant bands were compared to a 100BP DNA ladder to confirm their sizes. The PCR amplicons were sequenced at Inqaba Biotech Industries (Pretoria, South Africa) in a Sanger sequencer.

Trimming and alignment of sequence reads was performed in the Molecular Evolutionary Genetic Analysis v7 (MEGA 7) software (Center for Evolutionary Medicine and Informatics, Tempe, AZ, USA). Trimmed sequence reads were uploaded onto the Basic Local Alignment Search Tool (BLAST) program of the National Centre for Biotechnology Information (NCBI) and isolates identified by comparison with sequence reads available in the database. An E value of 0.0 and sequence of identity ≥ 99% was accepted as the minimum requirement for successful identification to genus or species level. All sequence reads have been deposited with the NCBI GenBank, under accession numbers MT367188 to MT367199.

### Biolog® MT2 microplate assay for screening isolates for MC-LR-degrading ability

The ability of individual bacterial strains to utilize MC-LR as a growth substrate was measured in the Biolog^®^ MT2 microplate assay. For the assay, cells were harvested from 24-h-old cultures, washed in PBS to remove traces of carbon, reconstituted in MSM and the optical density adjusted to 1.5. Bacterial cell suspensions were inoculated in wells spiked with MC-LR in MSM (pH 7) to a final concentration of 1, 10 and 100 µg L^−1^. Isolates were inoculated in triplicate, and two negative controls were set up: bacterial cells with no MC-LR, MC-LR in MSM with no bacterial cells. Plates were incubated at 30°C in the dark for up to 120 h. Measurements of optical density at 590 nm were taken in a VarioSkan Flash plate reader (Thermofischer Scientific, South Africa) at 0, 3, 6, 9, 18, 21, 24, 27, 30, 33, 48, 60, 72 and 96 h. In another experiment to investigate the effects of pH on MC-LR metabolism bacteria were grown in 10 µg L^−1^ MC-LR at pH 6, 8 and 9. Prior to data analysis, the absorbance values from each well were corrected by blanking against the corresponding absorbance at 0 h and negative values were adjusted to zero. Positive bacterial growth was denoted by a significant increase (ANOVA, *p* < 0.05) in optical density (OD_590_) in the experimental wells (1, 10 and 100 µg L^−1^ MC-LR), over the incubation period, as compared to the control wells (0 µg L^−1^ MC-LR).

### Screening isolates for the presence of the mlr gene cluster

For PCR screening, genomic DNA was extracted from 24-h-old cultures using the Zymo genomics Quick g-DNA extraction kit (Zymo Research Corporation, USA). Primer pairs used for the amplification of the four target genes (Table 2) were obtained from the literature (Saito et al. 2003). The PCR mixture (25 µL) consisted of 12.5 µL master mix, 1 µL each of forward and reverse primer, DNA template (50–100 ng) and nuclease free water added to make up to volume. PCRs were performed in a BioRad T100 thermal cycler using the cycling conditions listed in Table 3 (Dziga et al. 2012; Gandhi & Kumar 2017). Positive PCR amplification was confirmed with agarose gel electrophoresis.

**Table 2:** List of primers used for screening the *mlr* gene cluster

Gene	Primer name	Sequence	*Position	Tm (°C)	Size (bp)
*mlr A*	mlrAF 1	GAG CCG ATG TTC AAG ATA C	103–123	51.6	807
	mlrAR	CTC CTC CCA CAA ATC AGG	891–911	55.1	
*mlr A*	mlrAF 2	TCG CCA TTT ATG TGA TGG CT	457–477	55.7	453
	mlrAR	CTC CTC CCA CAA ATC AGG	891–911	55.1	
*mlr B*	mlrBF	CGA CGA TGA GAT ACT GTC C	99–117	52.0	448
	mlrBR	CGT GCG GAC TAC TGT TGG	530–547	55.9	
*mlr C*	mlrCF	TCC CCG AAA CCG ATT CTC CA	98–117	58.4	666
	mlrCR	CCG GCT CAC TGA TCC AAG GCT	744–764	61.8	
*mlrD*	mlrDF	GCT GGC TGC GAC GGA AAT G	51–69	59.8	671
	mlrDR	ACA GTG TTG CCG AGC TGC TCA	702–722	61.7	

*location on *Sphingomonas MJ-PV* strain

**Table 3:** PCR cycling conditions for *mlr* genes

Gene	Cycling conditions	No. cycles
*mlr* A	94°, 5 min; 94 °C, 30 s; 53°C, 30 s; 72°C, 1 min; 72 °C, 8 min; 4°C, ∞	30
*mlr* A	94°, 5 min; 94 °C, 30 s; 53°C, 30 s; 72 °C, 1 min; 72 °C, 8 min; 4°C, ∞	40
*mlr* B	94°, 5 min; 94 °C, 30 s; 53°C, 30 s; 72 °C, 1 min; 72 °C, 8 min; 4°C, ∞	40
*mlr* C	94°, 10 min; 94 °C, 20 s; 55 °C, 10 s; 72 °C, 30 s; 72 °C, 10 min; 4°C, ∞	40
*mlr* D	95°, 3 min; 95 °C, 30 s; 60 °C, 30 s; 72 °C, 1 min; 72 °C, 5 min; 4 °C, ∞	40

## Results

### Isolation and characterization of probable MC-LR degraders

A total of 17 isolates with different colony characteristics were isolated from oil refinery effluents (10 isolates) and tannery dumpsite soil (7 isolates), previously enriched in MC-LR. MALDI-TOF–MS biotyping classified the isolates into to three genera: *Bacillus*, *Paenibacillus* and *Lysinibacillus* (Table 4). Using this technique, 10 isolates were successfully identified to species level, 6 to genus level and 1 remained unidentified. The 16S rRNA gene was sequenced and used for isolate identification as well. The amplification of the 16S rRNA gene was not successful with three isolates (TL2, TL9, TL10 and TL17) as insufficient DNA could be extracted with both the heating method and with commercial DNA extraction kits. Therefore, the identity of these bacteria was inferred from MALDI-TOF-MS biotyping only. From phylogenetic analysis of sequence reads isolates were classified into three genera: *Bacillus*, *Paenibacillus* and *Lysinibacillus* (Table 4). There is high comparability between the results from MALDI-TOF–MS biotyper and 16S rRNA gene sequencing. For example, the isolates TL1 (*Bacillus subtilis*) and TL15 (*Bacillus cereus*) were identified to species level with the same identity in both techniques. In a similar manner isolates TL3, 5, 6 and 7 were all identified to genus level by both techniques. Discrepancies were noted with the identification of isolates TL4, TL8, TL11, TL12 and TL 13. Isolate TL4, 11, 12 and 13 were identified to species level using the MALDI-TOF-MS biotyper while with the 16S rRNA these were identified only to genus level. On the contrary, TL8 was identified to species level with the 16S rRNA gene sequencing and only to genus level with the MALDI-TOF–MS.

**Table 4:** Identity of bacteria isolated from oil refinery plant effluent and tannery dumpsite soils

Isolate code	MALDI-TOF MS identification		16S rRNA identification		
	*Identity	Score	*Closest match	% Similarity	Accession number
TL1	*Bacillus subtilis*	> 2	*Bacillus subtilis*	100	MT367188
TL2	*Bacillus* sp.	> 1.7	ND	**–**	**–**
TL3	*Lysinibacillus* sp.	> 1.7	*Lysinibacillus* sp ICMP 20,859	100	MT367189
TL4	*Paenibacillus glucanolyticus*	> 2	*Paenibacillus* sp. SP23 CP	99	MT367190
TL5	*Bacillus* sp.	> 1.7	*Bacillus* sp. C-1-21	100	MT367191
TL6	*Bacillus* sp.	> 1.7	*Bacillus* sp. CM-CNRG 437	100	MT367192
TL7	*Bacillus* sp.	> 1.7	*Bacillus* sp. CM-CNRG 437	100	MT367193
TL8	*Lysinibacillus* sp.	> 1.7	*Lysinibacillus boronitolerans*	100	MT367194
TL9	*Lysinibacillus fusiformis*	> 2	ND	**–**	**–**
TL10	*Bacillus cereus*	> 2	ND	**–**	**–**
TL11	*Bacillus cereus*	> 2	*Bacillus* sp. MPTDI	100	MT367195
TL12	*Bacillus cereus*	> 2	*Bacillus thuringiensis*	100	MT367196
TL13	*Bacillus cereus*	> 2	*Bacillus* sp. APB SML B20	100	MT367197
TL14	*Bacillus cereus*	> 2	ND	**–**	**–**
TL15	*Bacillus cereus*	> 2	*Bacillus cereus* P1035	100	MT367198
TL16	ND	< 1.7	*Lysinibacillus boronitolerans* P2IIIb	97	MT367199
TL17	*Bacillus cereus*	> 2	ND	**–**	**–**

*ND not determined

### Microcystin-degrading potential of isolates

**Fig. 2:**
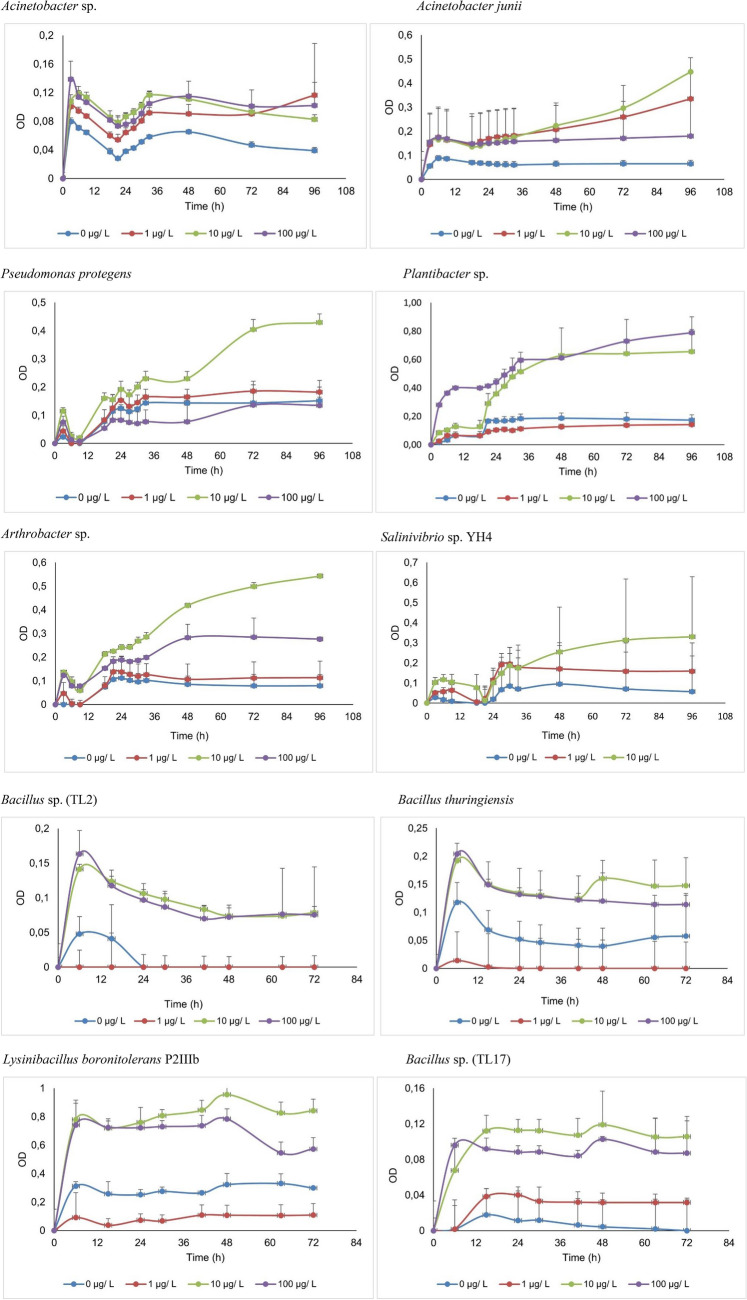
The growth patterns of bacteria (measured as optical density at 590 nm) grown in MC-LR enriched mineral salts media. Error bars represent standard deviation for three replicate values

A total of 32 bacterial strains obtained from saltpans, hot springs, car wash effluent, oil refinery plant effluent and tannery dumpsite soil were screened for their ability to metabolize the cyanobacterial toxin MC-LR in a calorimetric assay. The isolate *Bacillus* sp. (TL2) isolated from tannery waste dumpsite soil as well as *Bacillus thuringiensis, Lysinibacillus boronitolerans* P2IIIb and *Bacillus* sp. (TL17) (all isolated from oil refinery effluents) showed significant growth in the presence of MC-LR as a sole source of carbon and nitrogen. Six other bacterial isolates obtained from saltpans, hot springs and carwash effluents; *Acinetobacter* sp., *Acinetobacter junii*, *Arthrobacter* sp., *Plantibacter* sp., *Pseudomonas protegens* and *Salinivibrio* sp. also showed significant growth in the presence of MC-LR (Fig. 2). For these bacteria, there was a significant increase (ANOVA, *p* < 0.05) in optical density (OD_590_) in the experimental wells (1, 10 and 100 µg L^−1^ MC-LR), over the incubation period, as compared to the control wells (0 µg L^−1^ MC-LR). The patterns of growth of the different isolates show that the initial concentration of MC-LR affects the rate of growth of bacteria. For most isolates (*Plantibacter* sp., *Pseudomonas protegens, Salinivibrio* sp.*, Lysinibacillus boronitolerans* P2IIIb and *Bacillus* sp. (TL17)), growth was highest at 10 µg L^−1^ MC-LR, while for *Arthrobacter* sp. and *Bacillus* sp. APB SML B20 the growth was highest at 100 µg L^−1^. On contrary for *Acinetobacter* sp. and *Bacillus* sp. (TL2), growth was minimal at 1 µg L^−1^ and there was no significant difference between 10 and 100 µg L^−1^. On the other hand, for *Acinetobacter junii* growth was minimal at 100 µg L^−1^ and there was no significant difference between 10 and 1 µg L^−1^.

**Fig. 3:**
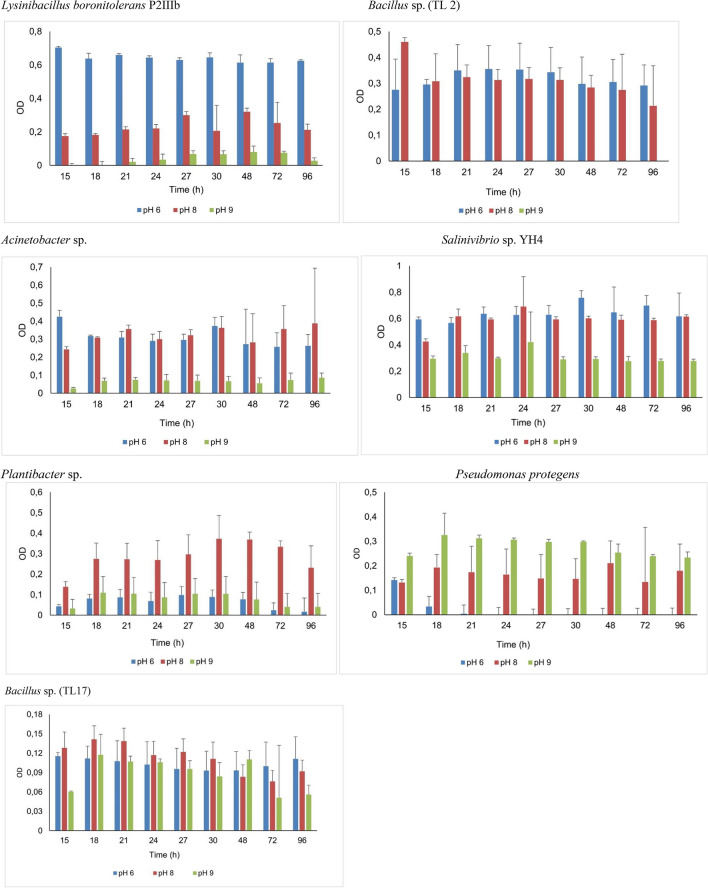
The growth (expressed as OD_590_) patterns of bacterial isolates grown at 10 µg L^−1^ MC-LR at pH 6, 8 and 9. The error bars represent standard deviation of three replicate analysis

The effect of pH on MC-LR metabolism was also evaluated by inoculating bacteria at different pH levels. The growth patterns of bacteria (Fig. 3) show that pH influences their patterns of growth, while *Pseudomonas protegens*, *Arthrobacter* sp, and *Plantibacter* sp. showed optimal or the highest growth under alkaline conditions (pH 8 and 9); *Bacillus* sp. (TL2) and *Lysinibacillus boronitolerans* P2IIIb grew optimally under slightly acidic conditions (pH 6) while for other isolates (*Salinivibrio* sp, *Bacillus* sp (TL17), *Bacillus thuringiensis* and *Acinetobacter junii*) the influence of pH was non-significant.

PCR was used to amplify *mlrA, mlrB*, *mlrC* and *mlrD* genes, which code for the enzymes responsible for MC-LR degradation and no PCR bands were formed in all 32 bacterial isolates, implying that the bacteria lacked the *mlr* genes.

## Discussion

A total of 17 isolates were obtained from oil refinery effluent and tannery dumpsite soil cultures previously enriched in MC-LR. Based on MALDI-TOF-MS biotyping and 16S rRNA gene sequencing the strains belonged to phylum Firmicutes under the genera: *Bacillus, Paenibacillus* and *Lysinibacillus*. While MALDI-TOF-MS biotyping is widely used in clinical microbiology, applications in environmental microbiology (especially extreme environments) remain limited because of a lack of robust and accurate reference spectra (Jang & Kim 2018). In this study there was homogeneity in the identification of bacteria using MALDI-TOF-MS biotyping and 16S rRNA gene sequencing. This is commensurate with other reports in which the technique was used successfully to identify environmental isolates (Kopcakova et al. 2014; Popović et al. 2017).

While extreme and polluted environments are presented as hot spots for biotechnologically viable microorganisms, they have not been explored as potential microcystin degraders (Sibanda et al. 2017). The isolated bacteria together with those obtained from saltpans, hot springs and car wash effluents were evaluated for MC-LR degradation. Normally, MC-LR degradation is evaluated through measuring the growth of bacteria in the presence of MC-LR as the only available source of nutrition. The Biolog MT2 microplate calorimetric assay, used in this study, is a rapid, low cost, versatile assay (Manage et al. 2009) that enables the use of considerably low concentrations of toxins (about 5 ng per isolate) (Idroos and Manage 2018). Of the 17 isolates obtained from tannery dumpsite soils and oil refinery effluent, 4 (*Bacillus* sp. (TL 2*)*, *Bacillus thuringiensis*, *Lysinibacillus boronitolerans* and *Bacillus* sp. (TL17)) could metabolize MC-LR in the Biolog MT2 plates. These isolates had previously been exposed to MC-LR, which could improve their MC-LR metabolizing potential (Ding et al. 2022). From saltpans, hot springs, and car wash effluent six isolates: *Acinetobacter* sp*.* DUT-2, *Acinetobacter junii*, *Arthrobacter* sp. B105, *Plantibacter* sp. PDD-56b-14, *Salinivibrio* sp. YH4 and *Pseudomonas protegens* could metabolize MC-LR as a growth substrate on the Biolog MT2 microplate assay*.* Notably, these had no previous exposure to MC-LR. In aquatic ecosystem, biodegradation of microcystins is the primary mechanisms by which they are detoxified and removed from the environment. The degradation is facilitated by heterotrophic bacteria that metabolize the toxins for their nutrition. Through enzyme activity, these bacteria cleave the bonds in the cyclic structure, breaking it into smaller straight chain compounds with less toxicity (Li et al. 2017). The ability of bacteria in this study to grow in the presence of MC-LR as the only source of carbon and nitrogen suggests that they can metabolize the toxin and can therefore be accepted as tentative MC-LR degraders.

Initial microcystin concentration and pH have been identified as some of the factors that affect MC-LR degradation (Li et al. 2017). In this study, initial MC-LR concentration and pH both affected the rate of MC-LR metabolism. Most of the isolate’s growth was significantly higher at 10 µg L^−1^, which could be accepted as the optimum MC-LR concentration at which metabolism occurred. The MC-LR concentrations tested in this study are environmentally relevant because they are common in some aquatic ecosystems. Therefore, for the bacteria to be able to utilize MC-LR within the tested range of concentrations (1 to 100 µg L^−1^) asserts their relevancy in MC-LR environmental bioremediation. The isolates *Plantibacter sp* PDD-56b-14 and *Pseudomonas protegens* showed alkali-tolerant properties: as they grew optimally at pH 8 and 9. During episodes of cyanobacteria blooms, the rate of photosynthesis becomes very high and subsequently the pH increases to levels as high as pH 11. Therefore, alkali-tolerant bacteria that can withstand the high pH possess a great potential in real life applications of MC-LR remediation (Dziga et al. 2013).

Several genotypes of microcystin-degrading bacteria have been identified; however, the degradation pathway facilitated by the *mlr* gene cluster remains the most studied. Hence, the *mlr* gene cluster is often used as a proxy for MC-LR degradation (Li et al. 2017). The bacterial isolates in this study were further screened for the presence of the *mlr* gene cluster using PCR. The PCR was negative; there were no bands formed, suggesting that the isolates lacked the *mlr* genes. Therefore, it can be assumed that they use a different mechanism for MC-LR degradation. Although the *mlr* genes have a wide environmental distribution and have been identified within different taxa, MC-LR degraders lacking this gene cluster are also known and are of interest because they could be degrading MC-LR in a more efficient pathway (Kormas and Lymperopoulou 2013).

MC-LR degraders have been isolated from a range of environments, and they fall within different taxonomic groups. The putative MC-LR degraders genera reported in this study are affiliated within taxa that are known to have MC-LR degradative properties as well as those with no known MC-LR degradative properties. The genera, *Arthrobacter*, *Pseudomonas, Acinetobacter* and *Bacillus* have some of their members identified as MC-LR degraders. For instance, members of the genus *Arthrobacter* with MC-LR-degrading potential include six strains; FN392690, FN392691, FN392693, FN392694, FN392695, and FN392696 which were isolated from water (Lawton et al. 2011; Manage et al., 2009b). Similarly, four members of the genus *Acinetobacter; Acinetobacter* sp. CMBD-2, *Acinetobacter* sp. WC-5 and *Acinetobacter guillouiae* A2, isolated from the water and sediments of Lake Taihu in China have MC-LR degradative abilities (Li et al. 2016; Li and Pan 2014). Interestingly, *Acinetobacter guillouiae* A2 and *Acinetobacter* sp. CMBD-2 have MC-LR-degrading and *Microcystis aeruginosa* lytic properties. Their dual mechanism of MC-LR bioremediation is attractive; hence, it would be interesting to also evaluate the algalytic properties of the *Acinetobacter* strains reported in this study. From the genus *Pseudomonas,* three *Pseudomonas aeruginosa* strains with MC-LR-degrading ability have also been isolated from a water purification plant, and sediments (Lemes et al. 2015; Li and Pan 2014; Lr et al. 1997). However, *Pseudomonas protegens* is reported for the first time in this study as a tentative MC-LR degrader. *Bacillus* spp. are renowned as champions of biotechnology, unsurprisingly several *Bacillus* spp with MC-LR-degrading potentials have previously been isolated. These include *Bacillus* sp. *EMB* isolated from soil and an algal heap as well as *Bacillus flexus* SSZ 01, *Bacillus* sp*.* AMRI03*, Bacillus cereus* and *Bacillus nanhaiensis* all isolated from lake water (Alamri 2010, 2012; Hu et al. 2011, 2012; Zhang et al. 2015). *Bacillus thuringiensis*, which can also degrade pesticides and poly aromatic hydrocarbons, is reported here for the first time as a potential MC-LR degrader (Ferreira et al. 2016).

There are no available reports on members of three genera *Plantibacter*, *Salinivibrio* and *Lysinibacillus* MC-LR degradative properties. However, the putative MC-LR degradation ability of these strains is not surprising as each of the three genera appear to have representative members with renowned degradative potentials (Amoozegar et al. 2008; Lin and Yokota 2006; Wang et al. 2009).

## Conclusions

Bacteria isolated from oil refinery plant effluents, tannery dumpsite soils, saltpans, car wash effluent and hot springs could grow in the presence of MC-LR as a sole source of carbon, concomitant with their ability to degrade MC-LR. Their growth characteristics indicate that for most of them growth occurs optimally at 10 µg L^−1^ and is minimal at 1 and 100 µg L^−1^ MC-LR. For some of the strains, growth was also affected by pH with optimal growth observed in alkaline conditions (pH 8 and 9). This is relevant as microcystins are prevalent in cyanobacterial bloom infested water bodies where the pH is alkaline most of the time. The study suggests that bacteria with MC-LR degradation are widely distributed and occur even in extreme environments. Nonetheless, future studies aimed at elucidating the mechanisms of MC-LR degradation, identification of degradation products as well describing the molecular pathways of degradation are necessary.

## Data Availability

Raw sequences generated in the present study have been deposited with NCBI GenBank under accession numbers MT367188 to MT367199.

## References

[CR1] Alamri SA (2010). Biodegradation of microcystin by a new Bacillus sp. isolated from a Saudi freshwater lake. Afr J Biotech.

[CR2] Alamri SA (2012). Biodegradation of microcystin-RR by *Bacillus flexus* isolated from a Saudi freshwater lake. Saudi J Biol Sci.

[CR3] Amoozegar MA, Schumann P, Hajighasemi M, Fatemi AZ, Karbalaei-Heidari HR (2008). Salinivibrio proteolyticus sp. Nov., a moderately halophilic and proteolytic species from a hypersaline lake in Iran. Int J Syst Evol Microbiol.

[CR4] Bouaïcha N, Miles CO, Beach DG, Labidi Z, Djabri A, Benayache NY, Nguyen-Quang T (2019). Structural diversity, characterization and toxicology of microcystins. In Toxins.

[CR5] Brito EMS, Piñón-Castillo HA, Guyoneaud R, Caretta CA, Gutiérrez-Corona JF, Duran R, Reyna-López GE, Nevárez-Moorillón GV, Fahy A, Goñi-Urriza M (2013). Bacterial biodiversity from anthropogenic extreme environments: a hyper-alkaline and hyper-saline industrial residue contaminated by chromium and iron. Appl Microbiol Biotechnol.

[CR6] Chakravorty S, Helb D, Burday M, Connell N, Alland D (2008). A detailed analysis of 16S ribosomal RNA gene segments for the diagnosis of pathogenic bacteria. J Microbiol Methods.

[CR7] Ding Q, Song X, Yuan M, Xu K, Huang J, Sun R, Zhang J, Yin L, Pu Y (2022). Microcystin-LR exposure enhances toxin-degrading capacity and reduces metabolic diversity of sediment microbial communities. Environ Pollut.

[CR8] Dziga D, Wladyka B, Zielińska G, Meriluoto J, Wasylewski M (2012). Heterologous expression and characterisation of microcystinase. Toxicon.

[CR9] Dziga D, Wasylewski M, Wladyka B, Nybom S, Meriluoto J (2013). Microbial degradation of Microcystins. Chem Res Toxicol.

[CR10] Emami K, Nelson A, Hack E, Zhang J, Green DH, Caldwell GS, Mesbahi E (2016). MALDI-TOF mass spectrometry discriminates known species and marine environmental isolates of seudoalteromonas. Front Microbiol.

[CR11] Ferreira L, Rosales E, Danko AS, Sanromán MA, Pazos MM (2016). *Bacillus thuringiensis* a promising bacterium for degrading emerging pollutants. Process Saf Environ Prot.

[CR12] Gagala, I., & Mankiewicz-Boczek, J. (2012). The Natural Degradation of Microcystins (Cyanobacterial Hepatotoxins) in Fresh Water - the Future of Modern Treatment Systems and Water Quality Improvement. *Polish Journal of Environmental Studies*, *21*(5), 1125–1139. http://apps.webofknowledge.com/full_record.do?product=UA&search_mode=GeneralSearch&qid=17&SID=2Dpi1I@5FplN7ld6JD4&page=1&doc=1

[CR13] Gandhi VP, Kumar A (2017). Isolation and characterization of microcystin degrading bacteria from holy ponds in India. International J Appl Sci Biotechnol.

[CR14] Hu L, Zhang F, Liu C, Wang M (2011). Biodegradation of microcystins by *Bacillus* sp. strain EMB. Energy Procedia.

[CR15] Hu L, Zhang F, Liu C, Wang M (2012). Biodegradation of microcystins by *Bacillus* sp. strain EMB. Energy Procedia.

[CR16] Idroos FS, Manage PM (2018). Bioremediation of microcystins by two native bacteria: *Bacillus cereus* and *Rahnella aquatilis*. Asian J Microbiol Biotechnol Environ Exp Sci.

[CR17] Jang KS, Kim YH (2018). Rapid and robust MALDI-TOF MS techniques for microbial identification: a brief overview of their diverse applications. J Microbiol.

[CR18] Kochhar N, Kavya IK, Shrivastava S, Ghosh A, Rawat VS, Sodhi KK, Kumar M (2022). Perspectives on the microorganism of extreme environments and their applications. Current Res Microb Sci.

[CR19] Kopcakova A, Stramova Z, Kvasnova S, Godany A, Perhacova Z, Pristas P (2014). Need for database extension for reliable identification of bacteria from extreme environments using MALDI TOF mass spectrometry. Chem Pap.

[CR20] Kormas KA, Lymperopoulou DS (2013). Cyanobacterial toxin degrading bacteria: who are they?. Biomed Res Int.

[CR21] Kumar P, Hegde K, Brar SK, Cledon M, Kermanshahi-pour A (2019). Potential of biological approaches for cyanotoxin removal from drinking water: a review. Ecotoxicol Environ Saf.

[CR22] Lawton LA, Welgamage A, Manage PM, Edwards C (2011). Novel bacterial strains for the removal of microcystins from drinking water. Water Sci Technol.

[CR23] Lemes GAF, Kist LW, Bogo MR, Yunes JS (2015). Biodegradation of [D-Leu 1] microcystin-LR by a bacterium isolated from sediment of Patos Lagoon estuary Brazil. J Venomous Anim Toxins Includ Trop Dis.

[CR24] Li H, Pan G (2014). Enhanced and continued degradation of microcystins using microorganisms obtained through natural media. J Microbiol Methods.

[CR25] Li H, Ai H, Kang L, Sun X, He Q (2016). Simultaneous *Microcystis Algicidal* and microcystin degrading capability by a single *Acinetobacter* bacterial strain. Environ Sci Technol.

[CR26] Li J, Li R, Li J (2017). Current research scenario for microcystins biodegradation—A review on fundamental knowledge, application prospects and challenges. Sci Total Environ.

[CR27] Lin YC, Yokota A (2006). Plantibacter auratus sp nov, in the family *Microbacteriaceae*. Int J Syst Evol Microbiol.

[CR29] Manage PM, Edwards C, Singh BK, Lawton LA (2009). Isolation and identification of novel microcystin-degrading bacteria. Appl Environ Microbiol.

[CR30] Mesbah NM (2022). Industrial biotechnology based on enzymes from extreme environments. Front Bioeng Biotechnol.

[CR31] Pearson L, Mihali T, Moffitt M, Kellmann R, Neilan B (2010). On the chemistry, toxicology and genetics of the cyanobacterial toxins, microcystin, nodularin. Saxit Cylindrospermopsin Marine Drugs.

[CR32] Pham T-L, Dang TN, Bui X-T, Chiemchaisri C, Fujioka T, Varjani S (2019). Microcystins in freshwater ecosystems:occurrence, distribution, and current treatment approaches. Springer Singapore.

[CR33] Pham TL, Utsumi M (2018). An overview of the accumulation of microcystins in aquatic ecosystems. J Environ Manage.

[CR34] Popović NT, Kazazić SP, Strunjak-Perović I, Čož-Rakovac R (2017). Differentiation of environmental aquatic bacterial isolates by MALDI-TOF MS. Environ Res.

[CR35] Raddadi N, Cherif A, Daffonchio D, Neifar M, Fava F (2015). Biotechnological applications of extremophiles, extremozymes and extremolytes. Appl Microbiol Biotechnol.

[CR36] Saito T, Okano K, Park HD, Itayama T, Inamori Y, Neilan BA, Burns BP, Sugiura N (2003). Detection and sequencing of the microcystin LR-degrading gene, *mlrA*, from new bacteria isolated from Japanese lakes. FEMS Microbiol Lett.

[CR37] Salwan R, Sharma V (2022). Genomics of prokaryotic extremophiles to unfold the mystery of survival in extreme environments. Microbiol Res.

[CR38] Selvarajan R, Sibanda T, Tekere M, Nyoni H, Meddows-Taylor S (2017). Diversity analysis and bioresource characterization of halophilic bacteria isolated from a South African saltpan. Molecules.

[CR39] Selvarajan R, Sibanda T, Tekere M (2018). Thermophilic bacterial communities inhabiting the microbial mats of “indifferent” and chalybeate (iron-rich) thermal springs: Diversity and biotechnological analysis. MicrobiologyOpen.

[CR40] Sheik CS, Mitchell TW, Rizvi FZ, Rehman Y, Faisal M, Hasnain S, McInerney MJ, Krumholz LR (2012). Exposure of soil microbial communities to chromium and arsenic alters their diversity and structure. PLoS ONE.

[CR41] Sibanda T, Selvarajan R, Tekere M (2017). Synthetic extreme environments: overlooked sources of potential biotechnologically relevant microorganisms. Microb Biotechnol.

[CR28] Takenaba S, Watanabe MF (1997) *Microcystin LR degradation by* Pseudomonas aeruginosa *alkaline protease*. Chemosphere 34(4):749–757 10.1016/s0045-6535(97)00002-7 9569941

[CR42] Wang CY, Hsieh YR, Ng CC, Chan H, Lin HT, Tzeng WS, Shyu YT (2009). Purification and characterization of a novel halostable cellulase from Salinivibrio sp strain NTU-05. Enzyme Microb Technol.

[CR43] WHO. (2020). Cyanobacterial toxins: microcystins. Background document for development of WHO Guidelines for drinking Water-quality and Guidelines for safe recreational water environments. http://apps.who.int/bookorders

[CR44] Zhang J, Shi H, Liu A, Cao Z, Hao J, Gong R (2015). Identification of a new microcystin-degrading bacterium isolated from Lake Chaohu, China. Bull Environ Contam Toxicol.

